# Arsenic species in weathering mine tailings and biogenic solids at the Lava Cap Mine Superfund Site, Nevada City, CA

**DOI:** 10.1186/1467-4866-12-1

**Published:** 2011-01-24

**Authors:** Andrea L Foster, Roger P Ashley, James J Rytuba

**Affiliations:** 1 U.S. Geological Survey, 345 Middlefield Rd., MS 901 Menlo Park, CA, 94025, USA

## Abstract

**Background:**

A realistic estimation of the health risk of human exposure to solid-phase arsenic (As) derived from historic mining operations is a major challenge to redevelopment of California's famed "Mother Lode" region. Arsenic, a known carcinogen, occurs in multiple solid forms that vary in bioaccessibility. X-ray absorption fine-structure spectroscopy (XAFS) was used to identify and quantify the forms of As in mine wastes and biogenic solids at the Lava Cap Mine Superfund (LCMS) site, a historic "Mother Lode" gold mine. Principal component analysis (PCA) was used to assess variance within water chemistry, solids chemistry, and XAFS spectral datasets. Linear combination, least-squares fits constrained in part by PCA results were then used to quantify arsenic speciation in XAFS spectra of tailings and biogenic solids.

**Results:**

The highest dissolved arsenic concentrations were found in Lost Lake porewater and in a groundwater-fed pond in the tailings deposition area. Iron, dissolved oxygen, alkalinity, specific conductivity, and As were the major variables in the water chemistry PCA. Arsenic was, on average, 14 times more concentrated in biologically-produced iron (hydr)oxide than in mine tailings. Phosphorous, manganese, calcium, aluminum, and As were the major variables in the solids chemistry PCA. Linear combination fits to XAFS spectra indicate that arsenopyrite (FeAsS), the dominant form of As in ore material, remains abundant (average: 65%) in minimally-weathered ore samples and water-saturated tailings at the bottom of Lost Lake. However, tailings that underwent drying and wetting cycles contain an average of only 30% arsenopyrite. The predominant products of arsenopyrite weathering were identified by XAFS to be As-bearing Fe (hydr)oxide and arseniosiderite (Ca_2_Fe(AsO_4_)_3_O_3_•3H_2_O). Existence of the former species is not in question, but the presence of the latter species was not confirmed by additional measurements, so its identification is less certain. The linear combination, least-squares fits totals of several samples deviate by more than ± 20% from 100%, suggesting that additional phases may be present that were not identified or evaluated in this study.

**Conclusions:**

Sub- to anoxic conditions minimize dissolution of arsenopyrite at the LCMS site, but may accelerate the dissolution of As-bearing secondary iron phases such as Fe^3+^-oxyhydroxides and arseniosiderite, if sufficient organic matter is present to spur anaerobic microbial activity. Oxidizing, dry conditions favor the stabilization of secondary phases, while promoting oxidative breakdown of the primary sulfides. The stability of both primary and secondary As phases is likely to be at a minimum under cyclic wet-dry conditions. Biogenic iron (hydr)oxide flocs can sequester significant amounts of arsenic; this property may be useful for treatment of perpetual sources of As such as mine adit water, but the fate of As associated with natural accumulations of floc material needs to be assessed.

## Background

Knowledge of arsenic (As) species in mine wastes and in mining-impacted areas is especially important in the heavily mined western foothills of the Sierra Nevada, California, because the recreational and residential development that has occurred in this region over the past decades has the potential to increase human and ecosystem exposure to inorganic As, a known carcinogen [1]. The main host of gold in this region is low-sulfide, quartz vein-hosted (i.e., "lode") deposits, which are also enriched in As [2]. Identification and quantification of As species in lode gold mine wastes is a critical step in a realistic estimation of health risks associated with increased exposures because (a) there is a wide range in solubility (a key factor in bioaccessibility and therefore bioavailability) among solid forms of As [3-5] and (b) the dissolved, inorganic forms of As pose a high cancer risk [6]. Arsenic contamination of historically mined areas is a problem across the Western U.S. [7], and is not limited to lode gold deposits, but also occurs in porphyry copper and other types of base metal deposits [8].

Although human exposure to As is likely to be elevated in residential developments built directly on As-rich mine wastes or near former industrial sites contaminated with As [7,9], significant exposures can also result from the dispersal of these materials. Ingestion of As in drinking water is recognized as the exposure route presenting the greatest risk to humans, and dispersal of As-rich mine wastes can accelerate geochemical and microbiological reactions that release arsenic to waters. However, additional exposure pathways can be very important in mining-impacted areas. These include inhalation/ingestion of mine waste particles (particularly among children), uptake of As from contaminated soils into home garden foodstuffs, dermal absorption of As from former tailings retention ponds now used for recreational purposes (e.g., swimming, boating), and consumption of fish from historic tailings retention ponds [6]. For all exposure routes, one of the key factors in the amount of As assimilated into the body is the partitioning of As between solid phases (food, soil, mine waste) and aqueous phases (natural waters as well as lung, gastric, and intestinal fluids). Both *in vitro *bioaccessibility and *in vivo *bioavailability studies of As in mine wastes demonstrate that these parameters vary as a function of the oxidation state and local coordination chemistry (i.e., the species) of As present [10-12].

X-ray absorption fine structure (XAFS) spectroscopy is one of the few techniques that can be used to identify and quantify metal and metalloid species in complex solid mixtures; the principles of the technique and its benefits/drawbacks have been described elsewhere [13-15]. Over the past decade, several XAFS spectroscopic studies have been performed on lode gold mining wastes from the Sierra Foothills. These studies indicate that the dominant As-hosting sulfide phase in northern and central Sierra foothills deposits is arsenopyrite (FeAsS), and in the southern foothills it is arsenian pyrite (Fe(As,S)_2_) [16]. However, the two minerals co-occur in many deposits. Arsenopyrite contains approximately 46 wt% As, whereas pyrites from the Sierra foothills vary in As concentration from trace amounts to approximately 10 wt% As. Although minerals containing reduced forms of As typically have low solubility, oxidation of reduced iron (Fe), sulfur (S), and As proceeds rapidly in the presence of ferric iron (Fe^3+^) and molecular oxygen (O_2_) [17]. In addition, batch and electrochemical studies demonstrate that the rate of oxidative dissolution of As-doped synthetic pyrite is greater than As-free pyrite [18,19].

Once released from primary phase(s), As can re-partition onto (or into) secondary phases by one or more of the following mechanisms: adsorption, co-precipitation, isomorphous substitution, and stoichiometric precipitation. Adsorption involves bonding between As oxoanions [arsenite = (H_3-n_AsO_3_)^n-3^, and arsenate = (H_3-n_AsO_4_)^n-3^] and particle (mineral or poorly-crystalline phase) surfaces via direct or indirect bonds. In mining environments, particles of Fe^3+ ^(hydr)oxide are typically the primary adsorbents of dissolved As [13,20-23]. Co-precipitation refers to the incorporation of As into the structure of a mineral during its formation [24]. Co-precipitation of As with Fe^3+ ^(hydr)oxide is very common in As-contaminated mining environments, and produces highly strained crystals whose characteristics differ from those formed in the absence of As [25-28]. Isomorphous substitution is distinguished from co-precipitation in that the As oxoanion substitutes in a specific site in the crystal structure, replacing an anion of similar dimensions (i.e., sulfate, phosphate). Isomorphous substitution (also called solid solution) may or may not be limited by crystal strain effects, depending on the mismatch between the substituted and the native elements. Jarosite (KFe_3_(OH)_6_(SO_4_)_2 _is a commonly occurring product of sulfide mineral oxidation into which arsenic can substitute to high concentrations [20,24]. Formation of stoichiometric arsenic precipitates (minerals or poorly crystalline phases) may occur if supersaturation with respect to these phases is reached. This condition is most commonly achieved in the vicinity of other mineral surfaces, where the solution chemistry is altered by sorbed molecules. As a result, arsenic precipitates are often seen as reaction rinds on other minerals rather than isolated particles. Examples of common secondary arsenic(V)-bearing phases found in mine wastes include scorodite (FeAsO_4 _·2H_2_O) and arseniosiderite (Ca_2_Fe_3_(AsO_4_)_3_O_2_·3H_2_O) [20,22].

Dissolved As can also be incorporated into microbial biomass and/or biogenic precipitates in close spatial association with microbial biomass by both biological uptake and chemical sorption processes. We showed in a previous paper that As accumulated in both algal biomass and biogenic Fe^3+ ^(hydr)oxide from Lava Cap Mine Superfund Site [29]. The latter contained more As than the former, with concentrations approximately 1000-fold higher than the surrounding waters, and 2-20 times higher than typical mine tailings from the site.

The cycle whereby reduced forms of arsenic are oxidized and liberated from primary sulfide phases only to be incorporated into oxidized, secondary phases such as Fe^3+ ^(hydr)oxides and sulfates, can also run in reverse: that is, under reducing conditions, oxidized forms of arsenic can be mobilized via reductive dissolution of As-bearing Fe^3+ ^(hydr)oxide and other secondary phases [5,9]. Depending on the geochemical conditions prevalent in the system, arsenic can remain in solution or be re-sequestered in secondary reduced phases such as green rusts and iron or arsenic sulfides [9].

Identification and quantification of As species by XAFS proceeds by two very different methods. The *ab initio *method, used in model systems and simple natural systems, employs theoretical phase and amplitude functions specific to a given absorber-backscatterer pair (e.g., As-O, As-Fe, As-S) to fit the unknown XAFS spectrum, providing information on the identity, number, and radial distances of atoms around an average As atom [24,30,31]. This approach works best in systems where there are just one or two distinct arsenic species present. The linear combination (LC) method is used to identify and quantify As species in heterogeneous matrices where more than two arsenic species may coexist. The LC method fits a linear combination of model As spectra representing known species to the unknown XAFS spectrum. While LC has been used successfully (along with geochemical data) to interpret the speciation of a wide variety of trace elements in natural systems [15,32-35], it provides no inherent constraints on the number of model compounds used in fits. In addition, the identity of the most appropriate model compounds can only be arrived by trial-and-error (comparing goodness-of-fit parameters).

A refinement in the LC approach to quantification of solid-phase trace metal species using XAFS spectra has been the use of principal components analysis (PCA) prior to LC analysis [36]. PCA is a multivariate eigenanalysis technique that is applicable to datasets that are approximately normally-distributed with variables that are linearly related [37]. Applied to XAFS spectra, PCA places needed restraints on both the number and identity of spectra to be used in LC fits. The operations of PCA express the original data matrix (in this case, a set of several XAFS spectra) as the product of two new matrices: the first matrix contains eigenvectors (components), which are mathematical constructs describing independent sources of variation within the dataset. The second contains eigenvalues, which express the relative variance in the experimental data matrix explained by each eigenvector. The maximum number of eigenvectors (components) extracted by PCA is equal to the number of columns *c *or rows *r *in the data matrix, whichever is smaller. A main goal of PCA is to distinguish *principal *components from *secondary *ones. Principal components (PC)s are those which: (1) account for the majority of set variance (usually >> 10% each); (2) are necessary for adequate reconstruction of all spectra in the set; (3) meet various empirical and statistical selection criteria [38].

Although components are mathematical constructs with no physical meaning, a key assumption for subsequent LC XAFS analysis the number of principal components is equivalent to the number of unique model spectra needed to fit the dataset. Selection of these model spectra from a spectral library (of more than 30 in this study) is assisted by target transformation, in which each potential spectrum is tested for its ability to be reconstructed by the principal components. Spectra having the lowest reconstruction residuals are the most likely candidates for use in LC fits [36,39].

A linear combination fit constrained by target transformation is a model-dependent analysis--that is, the results obtained depend on the models chosen for fitting. PCA additionally provides a model-independent, quantitative means of comparing the variance within a set of samples. By defining a coordinate space in which the x-axis is the product of the first eigenvector (**v**_1_, the first principal component) and its sample-specific first eigenvalues (w_1_,_i _), and y-axis is the product of the second eigenvector (**v**_2_, the second principal component) and its sample-specific second eigenvalues (w_2,i _), variance plots can be generated in which the proximity between any two points (samples) on the plot is directly proportional to their similarity [36]. Since the first and second components combined usually describe > 50% of the variance within the dataset, meaningful conclusions regarding sample groupings can often be obtained from this plot, prior to quantitative analysis by LC.

In this report, PCA of As-K edge XAFS spectra coupled to LC analysis was used to identify and quantify As species in ore, tailings, lake bottom sediment and biogenic solids from the Lava Cap Mine Superfund Site (LCMS) in Nevada County, CA. PCA was also applied to chemical datasets obtained for sediment and water samples with the goals of visualizing and quantifying the variance within those datasets, identifying which chemical parameters were most important in producing sample variability, and understanding correlations among the chemical parameters themselves. This information was used to deduce the main processes involved in arsenic attenuation and release at the LCMS site, and to identify areas of particular concern for mitigation or remediation of arsenic contamination at the site.

### Site and Sample Description

**Figure 1:**
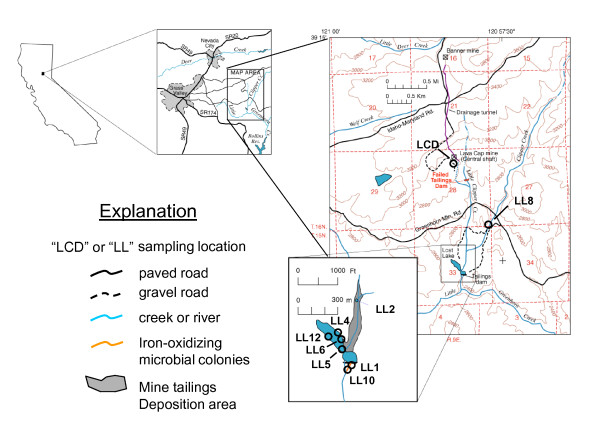
**Map of Lava Cap-Lost Lake study area, Nevada County, CA, with sampling locations indicated by black circles**. Base modified from U.S.Geological Survey 7.5-minute series Chicago Park quadrangle, 1979. See text for detailed site descriptions.

The LCMS site is located near Grass Valley, Nevada County, California, at an elevation of about 900 m (Figure 1). The mine exploited a quartz-carbonate vein system that was discovered in 1860. Early operations had limited success because the gold was dispersed in finer particles than was typical of deposits in the region. As summarized in Ashley [2], the mineralogy and geochemistry of the Lava Cap deposit is otherwise typical of the majority of low-sulfide gold-quartz deposits in the Sierra Nevada and elsewhere in the world.

During peak production years (1934 to 1943) about 90,000 tons of ore per year were processed at the mine [40,41], producing tailings (processed ore) requiring containment. The first tailings retention structure was a log dam located about 0.4 km below the mine on Little Clipper Creek (Figure 1; [42,43]). By 1938, it was at capacity, so a larger earth fill and rip-rap structure was constructed about 2 km farther downstream, which eventually came to be known as Lost Lake. During active mining, a slurry pipeline delivered tailings directly to the north lobe of Lost Lake. The Lava Cap Mine ceased production in June 1943 [44]. An estimated 1700 persons currently live within 1 km of the mine site and contaminated waterways [45].

In winter 1996 the upper half of the older log tailings dam collapsed, sending an estimated 7,646 m^3 ^of tailings into Little Clipper Creek, which discharges into Lost Lake [44,45]. During the time period covered in this study (1998-2002) tailings and waste rock covered about 8 acres of the mine property. By the end of 2007, stabilization of the remaining tailings on site and reconstruction of Little Clipper Creek where it passes through the mine site had been completed by the Environmental Protection Agency (EPA) and its contractors [45]. EPA is still finalizing its plan for management of contaminated surface and ground water [46].

Water is continuously discharged from the flooded mine through an adit (sample site "LCD" in Figure 1; LC1 in Table 1). The ore samples analyzed in this study (97-LCD1 and 97-LCD3 in Table 2) were collected from hand specimens found on the mine property near water site "LCD." Samples of Fe^3+ ^(hydr)oxide flocs and algal/cyanobacterial slimes (00-LC1-M1 and 00-LC1-M3 in Table 2) were collected from a small pond formed at the mouth of the mine adit (the pond has since been removed). Mine adit water joins Little Clipper Creek within about one hundred meters of its discharge point. Little Clipper continues for a few kilometers before joining Clipper Creek, which itself did not receive mine wastes or mine drainage. For this reason a site on Clipper Creek approximately 0.5 km upstream of its confluence with Little Clipper Creek was selected as the background site for water sampling; (LL8; Figure 1).

**Table 1:** Selected major and trace element dissolved constituents in water from several locations in the LCMS

Analyte	pH	**SpC**^**1**^	**DO**^**2**^	**Alk**^**3**^	Fe _total_	**Fe**^**2+**^	**Fe**^**3+4**^	**As**_**total inorganic**_	**As**^**3+**^	**As**^**5+ **^_**by difference**_	**As**^**5 **^**organic**	Ca	Cl	**SO**_**4**_
Units	-log[H+]	μS^6^	mg/L	mg/L	mg/L	mg/L	mg/L	μg/L	μg/L	μg/L	μg/L	mg/l	mg/L	mg/L
Detection Limit	±0.1	±1%	varies^7^	±5%	0.002	0.002	0.002	0.012	0.012	0.012	0.012	0.3	0.08	0.05
**LCD, Mine Adit**														
99	7.3	417	1	151	0.719	0.676	0.043	459	183	276	n.d	66.8	1.3	58
00	7.2	413	0.8	147	0.769	0.679	0.09	355	126	229	0.10	65.3	0.9	66
**Lost Lake Sites**														
**LL5**														
99	8.5	74.0	8	28.9	0.451	0.047	0.404	66.8	9.66	57.1	1.68	7.15	3.7	1.8
99-PW^8^	7.0	188	n.d.	83.7	10.7	7.02	3.68	3250	3250	0	0.95	26.3	6.5	0.6
00	9.4	43.1	8	12.6	0.088	0.01	0.078	17.9	1.31	16.59	1.51	2.90	3.3	1.4
00-PW	n.d.	164	n.d.	n.d.	0.845	0.605	0.24	45.1	25.9	19.2	0.76	22.0	5	10
00-R^9^	7.0	103	5	37.9	0.399	0.076	0.323	36.8	29.8	7.0	n.d	11.3	5	1.9
**LL6**														
99	8.6	71.0	8	27.9	0.401	0.026	0.375	86.7	16.2	70.5	1.68	6.8	3.6	1.6
00-R	8.5	95.6	7	40.1	0.51	0.039	0.471	44.1	5.90	38.2	1.32	8.2	4.8	1.3
99-PW	7.2	275	n.d	n.d.	7.01	5.53	1.48	31.3	36.7	0	5.33	n.d.	n.d.	n.d.
00-PW	n.d.	204	n.d	n.d.	0.923	0.877	0.046	38.1	26.5	11.6	0.58	31.0	4	9.3
**LL12**														
99	8.0	71.0	8	25.8	0.379	0.072	0.307	65.8	4.4	61.4	1.44	6.6	3.6	1.7
99-PW	7.0	285	n.d.	145	16.5	14.3	2.2	1580	2209^10^	0	0.65	32.4	7.1	0.5
00	7.1	39.9	n.d.	12.1	0.075	0.01	0.065	9.08	1.73	7.35	0.91	2.8	3.4	1.4
00-PW	n.d	412	n.d.	n.d.	7.80	7.429	0.371	1090	566	524	1.66	48.0	6.2	5.0
00-R	7.3	95.7	6	36.6	0.406	0.028	0.378	36.1	5.03	31.07	1.17	9.0	4.8	1.2
00-R-PW	7.7	339	1	164	0.859	0.854	0.005	2050	1880	170	1.70	34.0	7.5	0.6
**LL2, Pond In tailings**														
99	7.8	372	8	194	0.008	b.d	0.008	440	3.1	437	n.d	65.5	0.9	3.0
99-R	8.1	362	7	183	0.008	0.004	0.004	1340	6.1	133	n.d	59.5	1.0	1.9
00	7.6	374	7	199	0.018	0.008	0.010	614	4.38	609.62	3.11	59.1	0.6	2.0
00-R	7.6	362	7	182	0.009	0.005	0.004	533	5.64	527	0.74	62.7	0.7	2.3
**LL1 Seep at base of Lost Lake Dam**														
98	6.7	186	1	79.9	5.74	5.24	0.5	78.6	0.97	77.6	b.d	19.6	1.8	7.4
99	6.2	62	5	22.3	0.053	0.052	0.001	1.1	0.8	0.3	n.d	7.90	1.3	3.6
99-R	6.5	88	0.3	77.8	5.53	5.5	0.03	92.4	97.2	0	n.d	18.7	1.9	6.1
99-R2	6.5	178	0.4	78.8	5.15	4.51	0.64	92.4	77.9	14.5	b.d	19.2	2.2	6
00	6.7	198	0.1	89.1	6.251	5.345	0.906	61.8	69.6	0	0.07	20.5	1.7	9.4
00-R	6.6	188	0.2	78.5	5.77	5.78	0	85.1	85.0	0.1	b.d.	21.7	2.4	6.8
**LL10 approximately 75 meters downstream of LL1**														
99	7.0	158	5	71.1	1.77	1.56	0.21	43.2	42.4	0.8	b.d	19.7	2.2	5.8
**LL8 Background site upstream of mining impact**														
98	7.0	37.7	6	13.5	0.005	b.d.	0.005	0.098	0.025	0.073	b.d	3.4	1.6	2.3
99	6.5	38	8	12.8	b.d.	b.d.	b.d.	0.091	0.019	0.072	b.d	3.1	1.6	2.3
00-R	6.8	39.8	8	15.7	b.d	n.d.	n.d.	0.159	n.d.	0.159	b.d	3.3	1.8	2.2

Analytical methods are described in the text. Estimated lower limits of detection 5s method blank values are given for each analyte except pH, specific conductivity, and alkalinity (standard error given).^1 ^Specific conductivity; ^2 ^Dissolved oxygen; ^3^. Alk = alkalinity, caclulated as CaCO_3_; ^4 ^determined by difference; ^5 ^including mono-, di-, and tri-methyl arsenic compounds as well as other unidentified arsenic species; ^6 ^μS = microSiemens; ^7 ^± 0.1 when value is ≤ 1.0; ± 1.0 when value is ≥1.0; ^8 ^PW = pore water, extracted from sediment cores by centrifugation; ^9^R = repeat(not replicate) determination (same year, different collection date); ^10 ^Due to analytical error, As(III) values are occasionally greater than the total inorganic As value, especially when the proportion of As(III) is near 100%.

**Table 2:** Selected major and trace element constituents in sediments from several locations in the LCMS

Analyte	Al	Fe	P	S	As	Ca	Mn	Sb	Cd	Cu	Hg	Ni
Units	wt%	wt%	ppm	wt%	ppm	wt%	ppm	ppm	ppm	ppm	ppm	ppm
Detection Limit	0.005	0.004	10	0.01	2	0.013	0.71	±5%	0.69	1.4	0.01	0.26
**97-LCD1**^**1**^	1.79	0.99	50	0.61	1155	0.86	265	38	0.5	67	n.d	13
**97-LCD2**^**1**^	1.35	2.39	30	2.54	1130	0.71	295	2.4	9.5	9	0.4	11
**97-LCD3**^**1**^	1.52	2.39	b.d.	2.81	561	0.02	5	1	34.5	45	n.d	11
**97-LCD4**^**1**^	9.10	6.06	450	2.32	950	3.86	1290	3	0.5	88	0.02	2
**LC3 (n = 6) **^**1**^	5.13	2.26	b.d.	0.14	1043	3.10	731	16.12	5.17	35	1.01	18
**99-LL2-ss1**^**2**^	0.97	2.05	400	0.07	1640	1.76	590	n.r.	1.84	36.6	0.03	14
**99-LL12-1cs**^**1**^	7.10	4.16	70	0.45	1672	2.58	1030	15.2	6	60	0.82	34
**99-LL6-cs**^**1**^	5.82	3.40	60	0.5	2153	2.73	961	19	8	56	1.91	32
**00-LL5-cs**^**1**^	6.13	3.03	60	0.15	803	1.48	946	7.4	4	60	1.44	29
**99-LL4-ss1**^**2**^	0.99	1.78	420	0.07	514	1.68	615	n.r.	2.52	38	0.08	16
**99-LL4-ss2**^**2**^	0.76	1.54	380	0.07	546	1.77	470	n.r.	1.86	35.4	0.04	13
**99-LL10-m**^**2**^	0.32	15^3^	1810	0.05	5140	0.39	1040	4.1	0.5	18	b.d.	5
**00-LC1-m1**^**2**^	0.33	4.05	3650	0.97	8740	2.28	370	1.5	0.86	38.6	0.13	9.2
**00-LC1-m3**^**2**^	1.93	15^3^	4070	0.36	10,000^3^	1.9	920	15.3	9.25	485	5.14	43.6
**97-BCKGD **^**4**^	n.r.	n.r.	n.r.	n.r.	9.86	n.r.	n.r.	n.r.	2.06	62.8	b.d.	16.2

(ss), core samples (cs), microbial mats (m), and water filtrate (wf). Analytical methods are described in the text. Estimated lower limits of detection 5s method blank values are given for each analyte.^**1 **^Analysis by ICP-MS for a suite of 40 elements, HGAAS for As, Hg, and Sb, and LECO for S; ^**2 **^ICP-AES/MS of 32 elements;; ^5^Analyte value exceeds upper limit of detection. As a conservative estimate, the upper detection limit is reported as the table entry and used in PCA (see text); ^4 ^Analysis by ICP-AES (EPA method 6010).

The steep valley at the confluence of Clipper and Little Clipper Creeks contains an estimated 268,000 m^3 ^of tailings [44,45], and is called the "deposition area". Although these tailings were deposited over the entire history of mining at the LCMS; the older tailings are capped with recently deposited material released by the 1996 log dam failure (the approximate extent of this material is shown by the gray shading in the inset box in Figure 1). Water, sediment, and Fe^3+ ^(hydr)oxide flocs were sampled from a small, hyporheic zone water-fed pond in the deposition area (LL2; Figure 1). The pond is 2-3 meters deep and 3-5 meters in diameter, and in summer it has one or more blooms of algae and iron-oxidizing bacteria. The pond contains abundant aquatic plants and supports a population of frogs, despite its consistently high dissolved arsenic concentration (Table 1).

Lost Lake, the intact tailings retention structure, is between 4-12 m deep and covers approximately 20, 234 m^2 ^[46]. The lake is estimated to contain 115,000 m^3 ^of tailings, most of which would have been deposited in the 8-year period between its construction in 1934 and the cessation of large-scale mining in 1942 [46]. However, a blanket of tailings that washed in due to the 1996 dam failure presumably forms the uppermost layer of tailings in the bottom sediment. The lake takes on a greenish cast in summer, likely due to algal blooms. Debris from the 1996 tailings release initially blocked the flow of water over the dam spillway, resulting in a maximum temporary rise in lake level of approximately 1 m. When the blockage was cleared, the lake level quickly lowered, producing a "bathtub ring of very fine tailings deposited substantially above the water line. These materials were sampled several years later at site LL4 shown in Figure 1. Lake bottom sediments were cored with a hand-operated steel auger with polycarbonate sleeves at sites LL5, LL6, and LL12 (Figure 1). Pore water samples were also obtained from these sediment cores (LL5, LL6, and LL12 samples with the "PW" suffix in Table 1).

Water continuously seeps from the base of the intact rip-rap dam that forms Lost Lake, forming a small drainage that continues for 75-100 m before joining with surface water flowing over the concrete spillway. We previously reported that the near-neutral, Fe^2+ ^-rich, organic matter- and oxygen-poor seep drainage water upstream of the confluence supports the growth of a Fe-oxidizing microbial community whose main waste product, Fe^3+ ^(hydr)oxide, is an excellent sorbent for dissolved As [29]. Fe (hydroxide) flocs sampled at the mine adit (LCD) and from the Frog Pond (LL2) are also biogenic. Water and biogenic Fe^3+ ^(hydr)oxide were sampled at two sites below the dam. LL1 is < 0.5 m from the point where water emanates from the base of the rip-rap dam. Site LL10 is about 75 m downstream of LL1, just above the confluence of dam seepage water with Lost Lake spillway water.

## Results

### Variation in water and sediment chemistry

**Figure 2:**
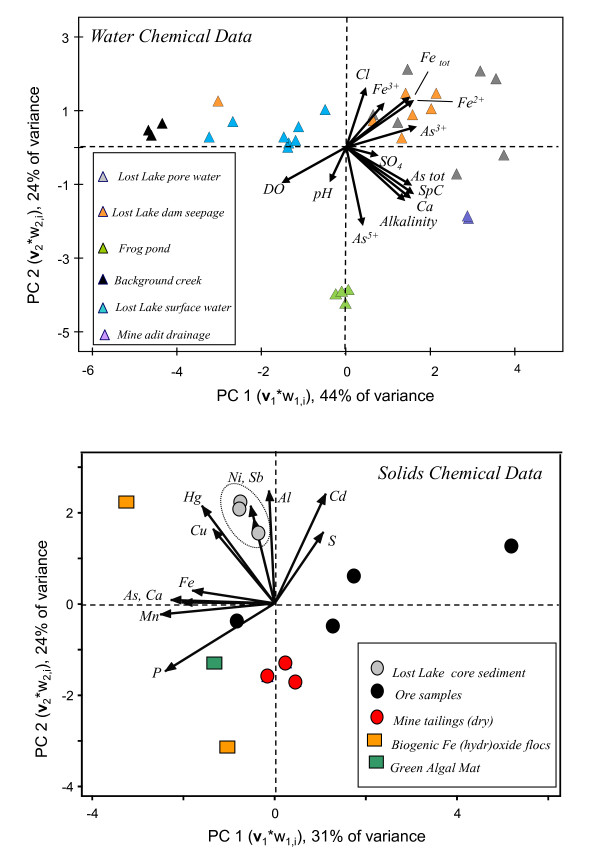
**Variance plots generated from principal components analysis (PCA) on selected water (top) and solids (bottom) chemical data collected at sites in the LCMS (see Tables 1 and 2)**. Solid arrows indicate the direction and importance of each of the chemical parameters to the sample distribution, and amount of parallelism of the arrows indicates the degree to which chemical parameters correlate.

The top panel in Figure 2 displays the results of a PCA based on selected water data. 68% of the total variance in the water dataset is described by axes (components) 1 and 2. The 12 water chemical variables are represented as vectors radiating from the origin of the plot. The degree of parallelism between two environmental vectors describes the strength of their correlation; for example, alkalinity and specific conductivity (SpC) are strongly correlated (r^2 ^= 0.82). These constituents are also closely correlated to total inorganic As (As_tot_) and Ca. Furthermore, the direction and magnitude of each vector indicates its importance as a constituent of the samples lying in the direction in which the vector points. For example, the vectors describing alkalinity, SpC, As_tot_, and Ca all point to the lower right quadrant of the graph; indicating that porewater and mine adit drainage water samples in that quadrant are elevated in these values relative to other samples.

Fe^2+^, Fe_total_, As^3+^, and Cl^- ^form another, less correlated vector cluster in the upper right quadrant of the graph, indicating that the Lost Lake pore water and Lost Lake seep water samples plotting in this quadrant are enriched in these constituents. The vector representing As^5+ ^points down in the direction of LL2 (Frog Pond) water samples, indicating their enrichment in this variable. Dissolved oxygen and pH vectors also point in the direction of the lower right quadrant, in which only LL2 samples plot. However, these variables do not point directly at other samples, but point in a direction between the surface water samples of LL2 and the surface water samples from LL8 (the background site) and Lost Lake (LL5, LL6, LL12), indicating that both types of water contain more oxygen and have slightly higher pH than other samples.

Examining PCA by sample type, indicates that the tightest groupings of water samples were site replicates (LL8, LL2, LCD-1); these were clearly separated from the mix of Lost Lake water sites (LL5, LL6, and LL12) as well as from the the dam seepage sites (LL1 and LL10). Lost Lake pore water samples were separated from the corresponding surface waters, and instead plotted nearest to dam seepage water samples (LL1 and LL10; Figure 2, top).

Surface water samples in the upper left quadrant of the graph (LL8, LL5, LL6, LL12) are high in dissolved oxygen (5-8 mg/l) and pH (6.2-8.5, median = 7.2, n = 12), but have lower values of other measured constituents relative to other samples. Average background site water samples (LL8; n = 3) were lowest in SpC (37.9 μS), alkalinity (13.2 mg/l), SO_4 _(2.3 mg/l), and As (0.1 μg/l;) of all the sites. This As value is about 100 times less than the maximum contaminant level (MCL) allowed for As (10 μg/l). Lost Lake surface water samples (n = 11) had higher values for these parameters than LL8 [SpC (71.0 μS), Fe (400 μg/l), SO_4 _(1.4 mg/l), alkalinity (27.9 mg/l), and As (40.5 μg/l, > 80% As^5+^)], but the values were lower than samples in the other 3 quadrants of Figure 2a. The mean As value in Lost Lake surface water was 45.4 μg/l, which is 10-100 times above the average background value and about 5 times the As MCL. There is one outlier in this quadrant, sample 99-LL1: as will be described, below, water chemistry of other samples from this site plot in a very different quadrant of the graph.

The primary difference between the surface water samples just described and surface water samples from LL2 (Frog Pond) in the lower-left quadrant (n = 4 ), is the amount of As^5+^. At 732 μg/l and > 90% As^5+^, the LL2 mean As value was roughly 1000 times above the average background value. Alkalinity and SpC had high values at site LL2 (188.3 mg/l and367 μS, respectively). Total dissolved Fe was low (8.8 μg/l), due to the high oxidation potential of the pond surface water.

The lower right quadrant of the graph contains samples from the mine adit LCD-1 (n = 2). Water from this site was high in SO_4 _(61.0 mg/l) and alkalinity (147.7 mg/l) relative to other sites. The median As value of 433 μg/l (59% As^5+^) was 1000 times above local background and about 50 times greater than the As MCL.

The diffuse cluster of samples in the upper right hand quadrant of the plot is composed of pore waters extracted from Lost Lake sediment cores (n = 7) and water seeping from the base of the intact dam forming Lost Lake (sites LL1 and LL10; n = 7). Mean values for dissolved Fe and As in Lost Lake pore water were 6,376 and 1,154 μg/l, respectively; inorganic As^3+ ^was clearly dominant in pore water, with an average relative abundance of 89.7%. The average concentration of Fe in seep water samples was moderately lower than lake porewater (4,323 μg/l), but significantly lower for As (64.9 μg/l). Arsenite (average of 82% As^3+^) was predominant in both water types, and the average pH of seep water (6.6) was the lowest of all water sample sites.

Another PCA was performed on the chemical data of ore, mill tailings, and biogenic solids listed in Table 2. The variance plot is presented as the lower panel in Figure 2. The two PC axes accounted for 55% of the total variance in the sediment chemical dataset. Although there are some sample clusters based on type (Lost lake core sediments = open circles, air-exposed mill tailings = open triangles), fewer replicates were available and only 2 obvious groupings are apparent within this dataset.

Phosphorous (P), manganese (Mn), calcium (Ca), As, and aluminum (Al) were the most important parameters describing the variance among solid samples. Manganese, Ca, and As are strongly correlated, while Al and P are not. All biogenic solids contain > 1000 mg/kg P, roughly 5 times more than tailings collected at the waterline or above the water line at Lost Lake or the Frog Pond (LL2; 300-400 mg/kg P), and 100 times that of tailings from Lost Lake sediment cores and most of the ore samples (< 100 mg/kg P; LCD-4 was the exceptional ore sample with 450 mg/kg P). Biogenic samples were highly enriched in As (7960 mg/kg; n = 3) relative to: (1) ore samples (949 mg/kg As; n = 4); (2) hand-augured mill tailings collected by split tube core in 1999-2000 from the scarp remaining after the log dam failure (1043 mg/kg As; n = 6); (3) tailings-rich Lost Lake bottom sediment (1543 mg/kg As; n = 3), and (4) subareally-exposed water-line tailings (530 mg/kg; n = 2).

Aluminum values varied widely in the hand-specimen ore samples, with argillite-rich ore samples having 5-9 wt% Al and the quartz-rich ore samples having only about 1.5 wt% Al (Table 2). Lost Lake bottom sediment samples have Al values comparable to argillite-rich ore (6.3 wt%; n = 3), but subareally-exposed tailings are much lower (0.9 wt%; n = 3). Two of the three biogenic solids had only 0.3 wt% Al (Table 2), and the relatively high Al content of the third LC1-M3 (1.93 wt% Al) may have resulted from entrainment of mineral particles within the biofilm sample collected for analysis.

Although not a predominant variable in the ordination, S values did follow trends with sample types: ore samples were the most enriched in sulfur (S), with concentrations > 2 wt% in 3 of the 4 ore samples. With the exception of LC1-M1, an algal mat sample with 1.0 wt% S, all tailings and biogenic solid samples contained less than 0.5 wt% S. The remaining elements in the ordination (Cd, Sb, Ni, Hg, and Cu) are considered chalcophilic elements, but only Cd is strongly positively correlated with S by the PCA (Figure 2, bottom).

### Variation in solid-phase arsenic speciation

**Figure 3:**
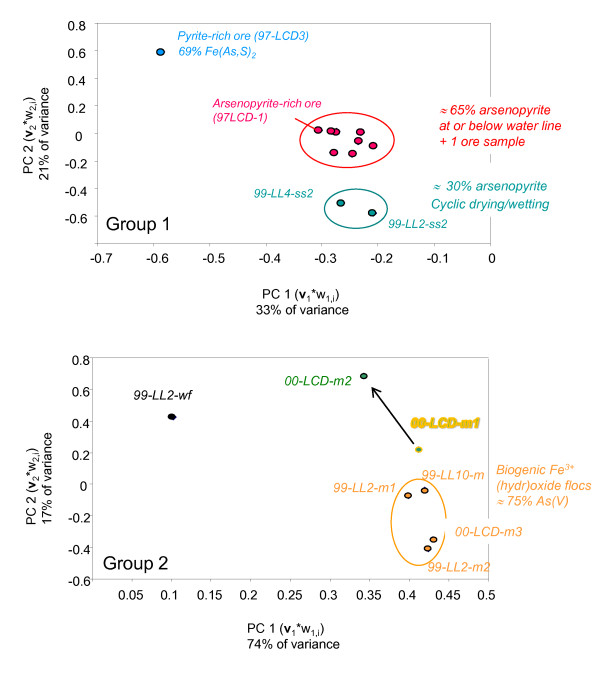
**Variance scatterplots of PCA results for Group 1 (ore, tailings, and Lost Lake sediments; top panel) and Group 2 (microbial mats/flocs; bottom panel), illustrating their utility as model-independent means of examining spectral variance**. Each point represents one sample EXAFS spectrum in a coordinate space defined by the product of the first eigenvector (principal component, **v**1) and its sample-specific first eigenvalues (w_1_,i ) on the x-axis, plotted against the product of the second eigenvector (**v**2) and its sample-specific second eigenvalues (w_2_,i ) on the y-axis. Axes are dimensionless. Outlying samples have been labeled for reference

**Figure 4:**
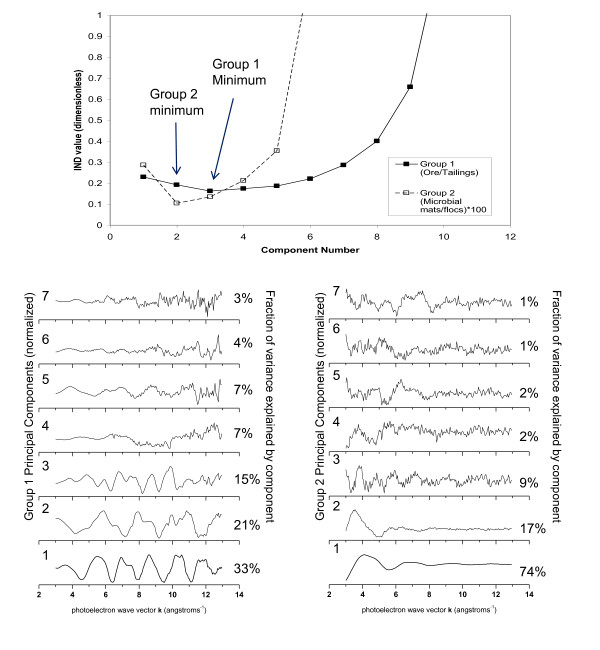
**(top) Malinowski indicator (IND) function plot for PCA of group 1 and group 2 EXAFS spectra (solid and dotted lines, respectively)**. The component number at which the function minimizes determines the transition from significant to secondary components, and suggests that 3 model As species are required for least-squares fits to group 1 EXAFS but only 2 model As species for group 2 fits. Visualization of the first seven normalized components from group 1 (bottom, left) and group 2 (bottom, right) confirms the IND function results: significant components have a high signal-to-noise ratio and each account for a significant percentage (ca. 5%) of the total variance within the group. Within group 1, PC4 and 5 are at the limits of empirical significance, which is discussed further in the text.

The top panel in Figure 3 displays the variance plot derived from a PCA on Group 1 EXAFS spectra (ore, tailings, and tailings-rich mud samples. It separated the spectra into three subgroups: the first contained the only sample of pyrite-rich ore (97-LCD3) and the second contained 2 samples of dry tailings (99-LL4-ss2 and 99-LL2-ss2). The third subgroup contained 8 samples: one ore sample (97-LCD1), 2 samples collected at the shorelines of Lost Lake (99-LL4-ss1) and Frog Pond (00-LL2-ss), and 5 subaqueous bottom sediment samples (99-LL2-ss1, 99-LL12-cs1, 99-LL12-cs2, 99-LL6-cs, and 00-LL5-cs). PCA on the 11 samples of Group 1 identified 3 definitive PCs (Figure 4, top panel). Visual analysis of the individual components (Figure 4, bottom left) suggests that the first 3 are clearly principal: together they accounted for a total of 69% of the variance within Group 1 and are clearly low in noise. Using the number of components required to reconstruct spectra as an indicator, 4 components are suggested, due to inability to reconstruct the features of spectrum LL5. Even when 4 components were used, spectral reconstructions did vary in quality among samples, with the single largest factor being the noise content of the unknown spectrum. Based on both pieces of information, the number of principal components in the group 1 PCA was determined to be 4. With the addition of the 4^th ^component, 76% of the spectral variance was accounted for.

The variance plot of the Group 2 PCA shows two clusters of closely related samples, and 3 individual samples that do not cluster (Figure 3, bottom). The isolated samples are: (1) water filtrate from Lost Lake (99-LL2-wf); (2) algal/cyanobacterial slime from a pool at the mine adit (00-LC1m1); and (3) the previous sample after a DI water rinse (00-LC1m2). The clustered samples are all biogenic iron (hydr)oxide rich flocs (Figure 3b); of these, samples of floc suspended in the water column (99-LL2-m1, 99-LL10 ) are separated along Axis 2 from floc samples collected from the sediment water interface (99-LL2-m2, 00-LC1-m3 ). The Malinowski IND function(see experimental methods section) for group 2 had a deep minimum at the second of the 7 PCs (Figure 4, top). Together PC1 and PC2 account for 91% of the variance in the dataset and contain signal as opposed to noise (Figure 4, bottom right). All spectral reconstructions in Group2 were adequate with just the two components identified by the IND values (reminder: Group 2 spectra were not k^3 ^weighted for PCA, due to high-amplitude noise).

Target transformations (TT) employing only the principal components from group 1 (n = 4) and group 2 (n = 2) were performed to identify the appropriate model spectra to be used in LC fits. From lowest to highest (best to worst), the TT reconstruction residuals from the group 1 PCA were: arsenopyrite (FeAsS) > As^5+^sorbed to Fe^3+ ^(hydr)oxide, > aqueous As^5+ ^> arseniosiderite (CaFe_3_(AsO_4_)_3_O_2_·3H_2_O) > amorphous ferric arsenate > arsenian jarosite [KFe_3_(SO_4_, AsO_4_)_2_(OH)_6_] > aqueous As^3+ ^> scorodite (FeAsO_4 _2H_2_O) > calcium arsenate (Ca_3_(AsO_4_)_2_) > arsenian pyrite [Fe(As,S)_2_]; (Figures. 4a and 4b). In the same order, the reconstruction residuals using the two primary components of the group 2 PCA were: As^5+^sorbed to Fe^3+ ^(hydr)oxide > arseniosiderite > aqueous As^5+ ^> amorphous FeAsO_4 _> aqueous As^3+ ^> K-jarosite > Ca_3_(AsO_4_)_2 _> scorodite >arsenopyrite > arsenian pyrite (Figure 4c). The two sulfide phases were nearly completely unreconstructed by the Group 2 PCs.

### Linear Combination Fits

#### Group 1

**Figure 5:**
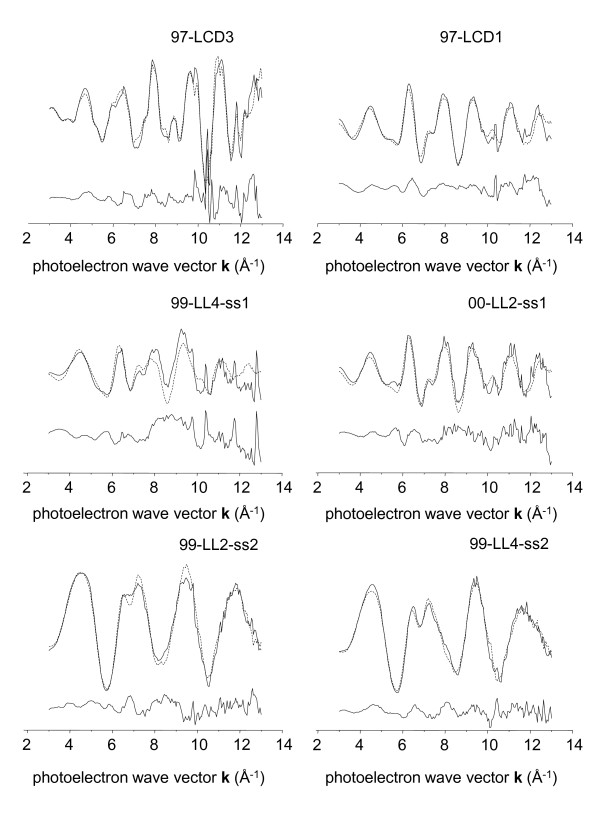
**Representative k^3^-weighted EXAFS spectra of ore and tailings samples from group 1 (solid lines), corresponding least-squares fits (dotted line), and fit residual (offset line)**. Some spectra contain crystal glitches that were removed for PCA.

**Figure 6:**
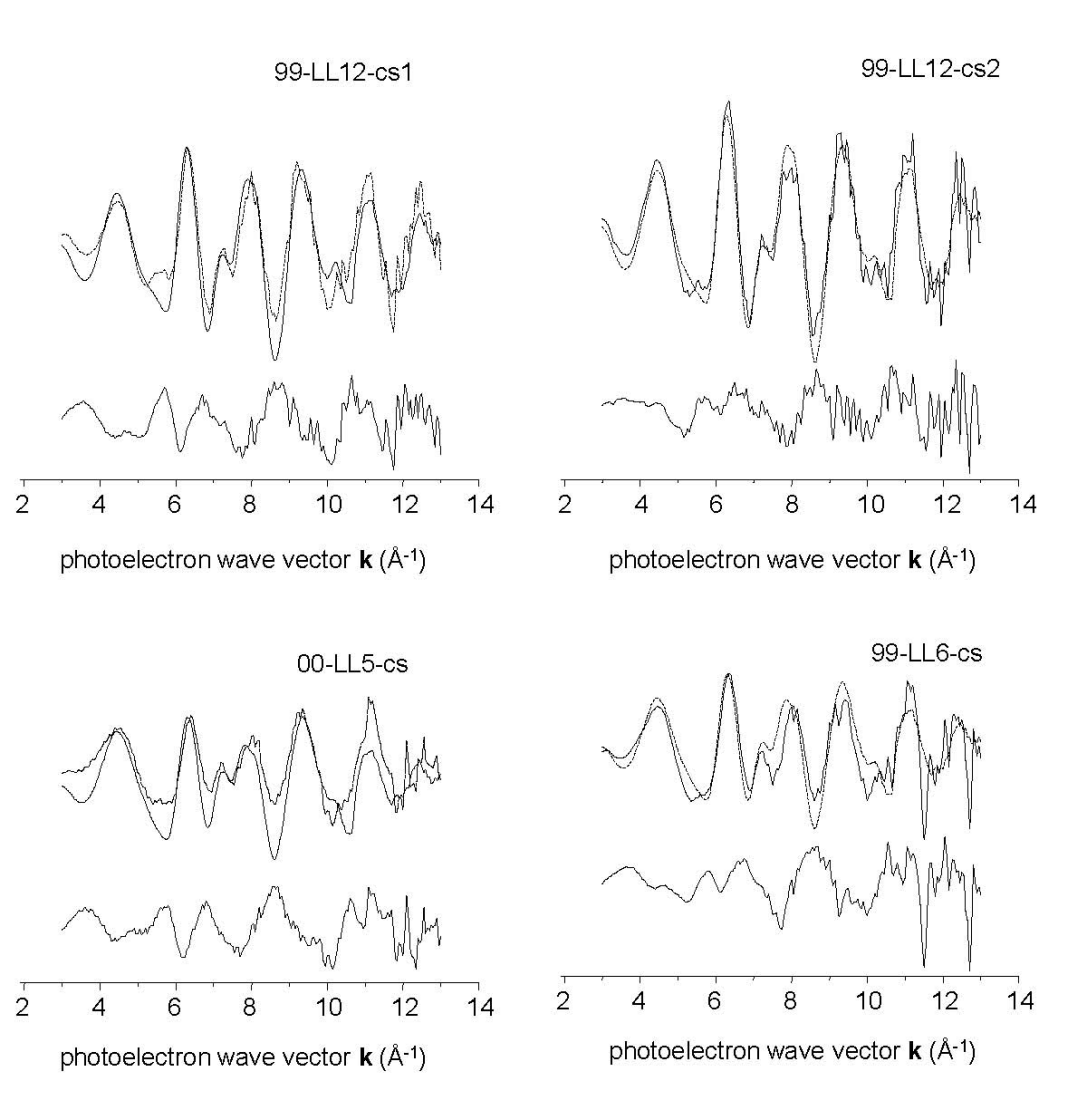
**Representative k^3^-weighted EXAFS spectra of Lost Lake bottom sediment core samples from group 1 (solid lines), corresponding least-squares fits (dotted line), and fit residual (offset line)**.

Initial fits were performed using the 4 model spectra with the lowest residuals in target transformation: for Group 1, these were arsenopyrite, As^5+ ^sorbed to Fe^3+ ^(hydr)oxide, aqueous As^5+^, and arseniosiderite. However, the contribution of the aqueous As^5+ ^spectrum to Group 1 fits was consistently < 0.01% of the total. The resulting three-component fits were adequate for all but one spectrum: 97-LCD3, which is known from SEM evidence to be an ore specimen unusually rich in arsenian pyrite (see SEM micrograph in Additional File 1). Based on these results, the aqueous arsenate spectrum was removed from the set of model compounds used in linear combination, least-squares (LCLS) fits and replaced with that of arsenian pyrite, even though its target transform residual was higher than several other model compounds (e.g., scorodite and calcium arsenate). The Group 1 spectra were re-fit, and the resulting k^3^-weighted spectra, fits, and fit residuals are displayed in Figure 5 and Figure 6. The fits confirm that arsenian pyrite was the predominant As species in 97-LCD3 (69 ± 10%), but was at the limit of significance (1-6%) in fits to all other samples in Group 1 (Table 3; Figure 5).

**Table 3:** Summary of linear combination, least-squares fits to EXAFS spectra

Sample	**Component (%)**^**1**^					Total	**χ2s**^**2**^
	FeAsS	**Fe(S,As)**_**2**_	**Arseniosiderite**^**3**^	**H**_**3**_**AsO**_**3 **_**(aq)**	**As(V)- Fe**^**3+ **^**(hydr)oxide**		
**Group 1- Ore and Mill Tailings samples**							
Ore samples							
97-LCD1	96	4	7	0	0	107	139
97-LCD3	33	69	0	0	2	104	804
Submerged tailings in lake or pond							
99-LL12-cs1	69	4	5	0	0	78	171
99-LL12-cs2	78	6	8	0	0	92	146
99-LL6-cs	61	5	12	0	0	78	355
00-LL5-cs	49	5	17	0	0	71	225
99-LL2-ss1	70	5	17	0	6	99	203
Sediment at water line, lake and pond							
99-LL4-ss1	56	4	26	0	0	86	587
00-LL2-ss	91	3	4	0	0	98	234
Subareal, near lake or pond					0		
99-LL2-ss2	15	1	33	0	39	88	78
99-LL4-ss2	45	1	27	0	37	110	60
**Group 2-Microbiological samples**							
99-LL10-m	0	0	0	34	71	105	114
99-LL2-m1	0	0	0	24	74	98	86
99-LL2-m2	0	0	0	19	76	95	76
99-LL2-wf	0	0	0	45	3	48	295
00-LCD-m1	0	0	0	60	60	120	137
00-LCD-m2	0	0	0	73	45	118	1408
00-LCD-m3	0	0	0	14	79	93	156

^1. ^Fit error per component is estimated to be 10%. ^2.^χ^2^is defined as the sum of squares of the residuals.. ^3^Ca_3_Fe_4_(AsO_4_)_4_(OH)_6_•3H_2_O.

Table 3 summarizes the results of our coupled PCA-LCLS analysis of arsenic speciation at the Lava Cap mine. Arsenopyrite was the predominant As species in most samples, including subaqueous tailings from lake and pond sites (65.4 ± 8.5%), sediments collected at the water line (73.5 ± 31%); and an arsenopyrite-rich ore specimen (97-LCD1; 96 ± 10%). Subareal tailings contained significantly less arsenopyrite than submerged tailings (30 ± 21%, n = 2, and 65 ± 11%, n = 5). Arseniosiderite was the second-most important component in fits to Group 1 spectra. It constituted only 7 ± 10% of the As in the 97-LCD1 ore specimen, but average values were 11 ± 5% (n = 5) in submerged tailings, 12 ± 5% (n = 2) in sediments/tailings at the water-line, and 30% ± 4% in air-exposed tailings (n = 2). Arsenate (As^5+^) sorbed to Fe^3+ ^(hydr)oxide was the third most important species determined in group 1 fits; it reached unequivocally significant levels (> 10%) only in subareal tailings samples. Within this subgroup, it constituted an average of 38% of the fit (n = 2).

LS fits were not constrained to equal 100%, so the degree of under or over fit (particularly the former) provides information on the degree to which the reference spectra used in LS fits capture all the species information in the experimental spectral set. The ore samples were very well fit, as indicated by average fit values of 105%, as were air-exposed sediments (average = 99 ± 21%). However, water-line tailings were an average of 8% underfit, and submerged tailings/sediment samples were an average of 20% underfit. Since the error of the least-squares analysis is approximately 10%, the degree of underfit to the submerged tailings/sediment samples appears significant, and suggests that another As species may be present.

#### Group 2

**Figure 7:**
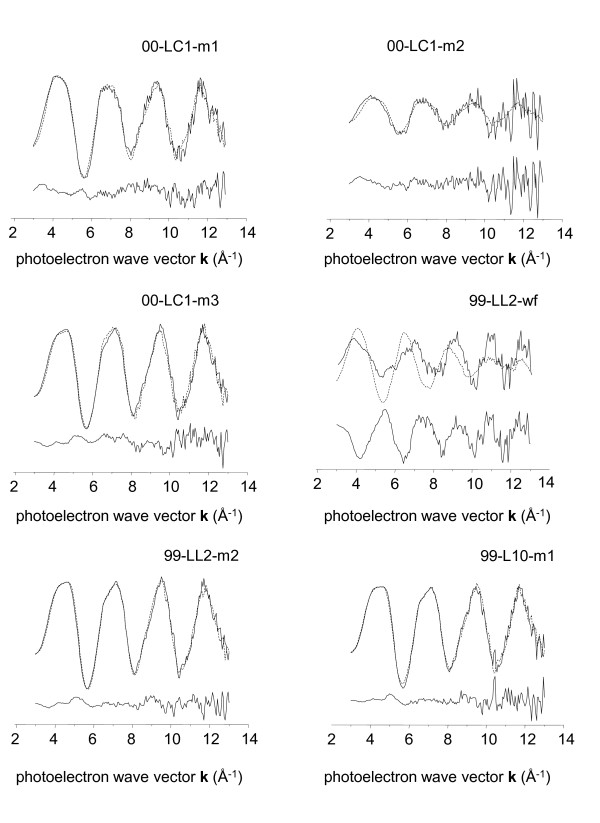
**Representative k**^**3**^**-weighted EXAFS spectra of group 2 microbial samples (solid lines), corresponding least-squares fits (dotted line), and fit residual (offset line)**.

Since PCA of group 2 indicated the presence of two primary components, LS fits to unweighted group 2 spectra initially used the two model compounds with the lowest reconstruction residuals in the TT analysis: As^5+^sorbed to Fe^3+ ^(hydr)oxide and arseniosiderite. However, these fits were not adequate to reproduce the entire set of spectra (not shown). For this reason, a trial and error approach was employed whereby suitability of each of the models was tested in two-component fits in which As^5+^sorbed to Fe^3+ ^(hydr)oxide was always one component (its magnitude was allowed to vary in fits). Only the addition of the aqueous As^3+ ^model compound made significant improvements in fit parameters, so this compound was used in conjunction with As^5+^sorbed to Fe^3+ ^(hydr)oxide to produce the fits reported in Table 3 and displayed in Figure 7.

Arsenic^5+ ^sorbed to Fe^3+ ^(hydr)oxide was the predominant species in fits to the 4 biogenic Fe^3+ ^(hydr)oxide flocs samples examined in this study. These samples are characterized by rust- or chocolate-brown Fe^3+ ^(hydr)oxide material. Arsenite (As^3+ ^oxoanion, in dissolved form) constituted 14 - 34% of the total arsenic in fits to XAFS spectra of biogenic Fe^3+ ^(hydr)oxide floc samples. Algal/cyanobacterial slime samples lower in Fe^3+ ^(hydr)oxide contained the most As^3+ ^of all the biogenic samples (Table 3). Frog pond water filtrate (99-LL2-wf in Table 3) was not adequately fit by any of the solid model compounds in our set; the most representative model available to us at the time of writing was aqueous As^3+^. We believe the most likely species is As3+ bound in biological tissues through sulfide-rich functional groups, based on analogy to previous studies [47].

## Discussion

### PCA and LCLS Fit Results

PCA was performed with the goal of determining the best reference spectra for solid-phase As species present in the LCMS as well as to identify differences among the samples. However, selection of reference spectra for use in LCLS fits by using target transformation (TT) residuals derived from group-specific PCs in some cases failed to select the most appropriate reference spectra to be used in fits. The "missing" species represented by these spectra (pyrite for group 1 and aqueous As^3+ ^for group 2) were of minor abundance in most samples, but were predominant species in outlier samples (LCD-1 in Figure 3, top and 99-LL2-wf in Figure 3, bottom). There could be several reasons for this. As discussed in [38], the TT residuals can be artificially increased due to several factors including small differences in calibration, resolution, and spectral noise among samples and models at different beamlines or data collection periods, and correlation between targeted vectors due to the oblique rotation inherent in target transformation. Another problem we encountered was in the ranking of spectra based on the TT residuals: similar reference spectra had similar TT residuals, yet only one of these similar spectra was needed to represent a particular species. If the spectrum needed to represent a very dissimilar species was ranked lower than the similar spectra, it would not be among those chosen for LC fits. However, trial-and-error fits did reveal the best spectra for LCLS fits described for Group2.

Several of the spectra were poorly fit despite all procedures to identify best matching model compounds (Figure 6 and Figure 7; Table 3). This is a likely indication that important species were missing from the library of model compounds used in this study. The missing species could constitute as much as 20% of the total arsenic present in Lost Lake bottom sediment samples and up to 52% of the Frog pond water filtrate sample 99-LL2-wf, which have the lowest fit totals. Inclusion of an additional spectrum of reduced As such as As^3+^sorbed to Fe^3+ ^(hydr)oxide, As^5+ ^associated with green rust, or arsenite adsorbed on pyrite or other sulfide may have been warranted in fits to Lost Lake bottom sediment, as has been the case in previous studies. However, arsenic sulfides (As_2_S_3_, crystalline and amorphous) were *not *detected in Lost Lake bottom sediment, indicating that if they are present, they constitute less than 10% of the total As. Inclusion of an As^3+^-organosulfur and/or As^5+ ^moiety such as As-glutathione may have improved the fit to the water filtrate sample (99-LL2-wf) as it did for natural algal biomass in a previous study [47].

### Predominant geochemical processes and implications for mass transfer of arsenic

Although EPA halted the further movement of As-rich tailings from the failed dam and the mine site several years ago, movement of dissolved arsenic through and off the site is still a concern. Water from the mine adit is a near-constant source of dissolved arsenic to the LCMS study area (at the time of this writing, the Environmental Protection Agency (EPA) was still working on its final remedy for contaminated areas and its plan for treating the mine adit water in perpetuity [46]). Monitoring of Little Greenhorn Creek (recipient of the majority of runoff from the LCMS; Figure 1), indicates that As levels are only slightly above the current MCL [45]. The fact that little impact of As from the LCMS is observed in downstream surface waters suggests that one or more of the following processes are occurring: (1) dilution of As-rich water by low-As water from Clipper Creek; (2) mass transfer of dissolved As offsite via ground water; and (3) sequestration of dissolved As in solid forms. The data gathered in this study do not provide significant insight into processes (1) and (2), but are useful in addressing process (3); this will be the focus of the discussion to follow.

The high Eh, SO_4_, SpC, Fe, and As values, near-neutral pH, and low DO of the mine adit water (LCD) support the conclusion that oxidation of iron- and arsenic-bearing sulfide minerals in a well-buffered geochemical environment is the process leading to enrichment of dissolved As (Table 2). The dataset used in this study was not extensive enough to explore seasonal variations in adit water chemistry and arsenic concentration, but it was noted in a previous investigation that winter rains generally dilute all constituents, including As [46]. The outlier LL1 value in Figure 2a may represent analytical error(s) or may actually be indicative of substantially different geochemical regime (for example, a period of high rainfall with substantial flushing and oxygenation) at the site during that sampling period.

Biogenic slimes and Fe^3+ ^(hydr)oxide flocs in a small pool at the adit outlet (LCD) were effective sorbents of As: they had the highest dry-weight values of arsenic of all solids in this study (Table 2). A considerable amount of As^3+ ^is retained by algal/cyanobacterial green slimes (Table 3); as in a previous study, most of the As^3+ ^is likely bound internally [47]. The algal/cyanobacterial slimes are anchored to rocks and not easily moved, but the Fe^3+ ^(hydr)oxide floc material is suspended in the water column, and could easily be washed downstream during winter storm events.

Previous work has demonstrated that attenuation of dissolved As (and other metals) occurs in Little Clipper Creek between the mine site and the confluence with Clipper Creek at the deposition area (the large deposit of tailings that extends out into the Lake, creating its northern and southern lobes; see gray area in Figure 1) [45]. Adsorption of As to mineral surfaces has been suggested as the cause of this attenuation [46], and this process is likely to occur in all the surface water bodies at the LCMS. We previously reported that arsenic is sequestered by algal/cyanobacterial and iron-oxidizing communities in water bodies of the LCMS [29], but the relative importance of abotic and biotic sequestration mechanisms has not been investigated. Substantial accumulations of Fe^3+ ^(hydr)oxide floc has been observed in Clipper Creek just below its confluence with Little Clipper. Based on our work with similar material at the seep sites (LL1, LL10; discussed below) this material is likely to be enriched in As. We have observed directly that the floc at this location is washed downstream during rain events; the implication is that any arsenic associated with this material would also be transported.

Net arsenic accumulation in the solid phase is also occurring in the bottom sediments of Lost Lake and Frog pond (LL2). Bottom sediments at both localities are predominantly composed of mill tailings, but contain about 1.5 times more arsenic than subareal tailings and ore samples examined in this study (Table 2). Although Lost Lake surface water was only slightly enriched in As, pore water samples had 2-10 times more As than mine adit water. Frog pond had very high concentrations of As in surface waters (roughly equal to adit water; Table 1). Diffusion of arsenic into the overlying water column as well as subsurface transport of As likely accounts for the dissolved As concentrations in Frog pond surface water, as it receives no surface water flow except during heavy rain events. Evapoconcentration in the dry summer months further increases the As concentration in Frog pond surface water.

The process of bottom sediment enrichment in Frog pond and Lost Lake likely begins with sorption of dissolved As onto biotic and abiotic particles as well as uptake of dissolved As by water-column biomass such as algae [47] and movement of arsenic through the aquatic food web. The high fractions of organoarsenic species in Lost Lake and LL2 pond water samples support this contention (Table 1), as does the unidentified As^3+ ^species in the water filtrate from Frog pond. Eventually, suspended organic and inorganic particulate detritus settles to the bottom and is incorporated in the sediments. Arsenic concentrations in pore water gradually rise owing to the remobilization of a fraction of the particulate arsenic in bottom sediment. Remobilization of particulate-associated arsenic can occur by several processes: (1) As associated with particulate organic matter (POM) can be remobilized by benthic biota, which ingest and assimilate it [48]; (2) As associated with biogenic Fe^3+ ^(hydr)oxide flocs (or with other Fe^3+^-bearing phases) can be remobilized by microbially-mediated reductive dissolution; (3) As can be released from sulfide minerals via oxidative dissolution or ligand-promoted dissolution (under reducing conditions).

The water data collected do not provide information on the importance of benthic biota to As remobilization from POM, but low Eh and SO_4 _values in Lost Lake pore waters coupled with their high SpC, Fe, and As (As^3+^) values are consistent with both the reductive dissolution and ligand-promoted dissolution hypotheses. However, the average relative abundance of arsenopyrite is higher in bottom sediments from lake and pond sites than it is from water line or subareal sites, suggesting that ligand-promoted sulfide dissolution is not the predominant process. Since there was presumably some oxidation of arsenopyrite prior to deposition, preferential dissolution of these oxidized products could produce the observed relative increase in the abundance of arsenopyrite determined by XAFS analysis in Lost Lake bottom sediment samples.

XAFS spectroscopy suggests that arseniosiderite (CaFe_3_(AsO_4_)_3_O_2_·3H_2_O) is a significant reservoir of oxidized As in submerged and water-line LCMS tailings, and that arseniosiderite and Fe^3+ ^(hydr)oxide are important As-bearing phases in subareal tailings. The data further suggest that arseniosiderite is more abundant as an oxidation product in dry tailings rather than submerged or water-line tailings (Table 3). The presence of arseniosiderite in LCMS tailings samples has not been confirmed by conventional X-ray diffraction or electron microbeam techniques, but precedent for its presence does exist in the literature. Arseniosiderite has been observed as a microscopic alteration product of loellingite (FeAs_2_) in "highly calcareous" samples [49] and has been identified by electron microbeam as an alteration product of arsenopyrite in Canadian gold mine tailings [20]. It has also been proposed to form nanoparticles during co-precipitation of Ca and As along with Fe^3+ ^(hydr)oxide, based on spectroscopic and microprobe data [20]. Arseniosiderite has been identified as a secondary phase at a French industrial site in which arsenopyrite was stockpiled [50]. It has also been observed under SEM in mine wastes from low-sulfide gold deposits in the Mojave Desert (J. Rytuba, pers. Comm.).

Arseniosiderite is a phase for which little thermodynamic data are available [20]. Determination of its stability under oxidizing and reducing conditions typical of LCMS would be beneficial to any analysis of arsenic mitigation strategies at the site. Krause and Ettel [51] reported the solubility of arseniosiderite to be 6.7 mg/l at pH 6.8 and 25°C. We converted the solubility expression for arseniosiderite to a molar solubility expression and calculated the saturation state of the water compositions listed in Table 1. The derivation and saturation state calculations are available in Additional File 2. No temperature compensation or activity corrections were applied to the data for these calculations. Our calculations indicate that mine adit water and most of the Lost Lake surface and water samples are saturated with respect to arseniosiderite (10 out of 13 samples for which all data were available to make calculations). Frog Pond (LL2 and seep (LL1, LL10) samples are predominantly undersaturated, with only one sample from each location exhibiting saturation. Although saturation indices derived in this way are approximations, the results largely support the solid phase speciation derived from LCLS fits, in which arseniosiderite was found only in the sediment from Lost Lake, Frog Pond, and subareal mill tailings. Once formed, arseniosiderite is probably not a permanent repository for As, because iron reducing bacteria are capable of deriving energy from the reduction of Fe^3+ ^in arsenate minerals just as they do from Fe^3+ ^in (hydr)oxides [52].

The seepage site at the base of Lost Lake dam is a location clearly dominated by biologically-mediated oxidation of dissolved Fe^2+^, formation of biogenic Fe^3+ ^(hydr)oxide, and sorption of arsenic. For about 100 m downstream of the seep issue point, between sampling points LL1 and LL10, the entire waterway is clogged with flocculent, biogenic Fe^3+ ^hydroxide whose dry weight arsenic concentrations approximately double that of LCMS mill tailings (compare values in Table 2). Seep water is presumed to have migrated through the extensive package of As-rich tailings that underlie Lost Lake and the deposition area prior to its issue point. The water composition is consistent with this interpretation, as it is suboxic, near-neutral in pH, contains high dissolved Fe^2+ ^and As.^3+ ^With the exception of the mine adit, the seep sites (LL1, LL10) have the highest sulfate values of all sites. The seep area is in a deep valley protected by trees, so only storm events generate enough energy to wash substantial amounts of this biogenic Fe^3+ ^(hydr)oxides downstream. However, reductive dissolution of this material by iron-reducing bacteria has the potential to introduce a new pulse of arsenic to the system. We have observed at least 3 feet of compacted Fe^3+ ^(hydr)oxide in hand-augered cores in and adjacent to the drainage formed by seep water.

## Conclusions

As residential development continues to encroach on rural lands previously used for mining, contact between humans and potentially toxic elements in solid and dissolved forms will increase. At the LCMS, as at other sites in the western U.S., there are large volumes of As-rich material which are economically impractical to treat for As removal or offsite disposal in a hazardous waste landfill. Onsite methods of sequestering the material and of preventing As from migrating offsite must be sought. Our analyses suggest that arsenopyrite, the primary As-bearing phase in Lava Cap mine tailings, can be stabilized if kept submerged in low oxygen water, as is currently the case for Lost Lake bottom sediments. XAFS analysis indicates that solid phase As^5+ ^is primarily associated with biogenic Fe^3+ ^(hydr)oxide and arseniosiderite. Arsenic associated with oxidized, fairly soluble phases can be immobilized by preserving the material under oxidizing, dry conditions. If this material is buried or submerged, however, microbially-mediated reductive dissolution of the Fe^3+^-bearing phases could result in re-mobilization of As.

## Experimental Methods

### Water and sediment chemistry

In the sections to follow, use of product names does not constitute endorsement by the U.S. Geological Survey. Field measurements of pH and Eh were collected using an Orion 290 meter and Ross triode or platinum electrode, respectively. Standard pH buffers of 4, 7, or 10 were used for two-point pH calibration and quinhydrone-saturated pH 4 and 7 buffers were used for Eh calibration. Dissolved oxygen was estimated in the field using a commercially available colorimetric method (Chemets ampoules). Alkalinity measurements were performed by Gran titration (using sulfuric acid) on 0.45 μm-filtered samples preserved on ice. Conductivity was determined using a portable meter (Orion or Corning) and appropriate standards. Major anions (Cl, SO_4_) were determined by ion chromatography on unpreserved, 0.45 μm-filtered samples (Ocala Labs, FL). Total inorganic As, inorganic As^3+ ^and organoarsenic species (mono- and dimethyl arsenic, trimethyl arsenic, and other unidentified compounds) were determined directly using hydride generation-cryotrapping-gas chromatography atomic absorption spectrometry (HG-CT-GC-AAS) on 0.45 μm filtered samples preserved with ultrapure HCl and refrigerated prior to analysis (Frontier Geosciences, Seattle, WA, or USGS laboratories). Dissolved inorganic As^5+ ^was determined by difference of inorganic total As and inorganic As^3+^species. Total dissolved Fe and dissolved Fe^2+ ^species were determined by the ferrozine spectrophotometric method (Frontier Geosciences, Seattle, WA, or USGS laboratories) on samples collected into amber bottles, preserved with ultrapure HCl, and kept under refrigeration until analysis; Fe^3+ ^was determined by difference.

Solids were collected into acid-washed jars, and major and trace elements determined by ICP-AES/ICP-MS on aqua regia extracts of air-dried material (Chemex Labs, Sparks, NV). Microbial mats were collected into sterile plastic jars or sterile syringes and stored on ice for transport to the laboratory. Mat material for chemical analysis was air dried, then major and trace elements were determined on aqua regia digests (Chemex Labs, Sparks, NV). Due to the high water content of several mats, there was an insufficient quantity of air-dried material for chemical analysis.

Contract and internal USGS analytical laboratories performed data validation using internal standards, reagent blanks, and reference samples per each lab's standard procedures. The USGS laboratories standard quality control criteria for accuracy was ± 15% of the expected value at five times the lower limit of detection and the accepted range of precision was ≤ 15% of the replicate mean value.

PCA of water and solids data was performed using PC-ORD v.5 [37]. PCA of chemical data was similar to that described in the introduction for XAFS spectra, with the following differences. Matrices with samples in *r *rows and environmental variables in *c *columns were generated for water (31 × 13) and sediment (13 × 12) data. Values below detection limit were assigned a non-zero value 10 times less than the detection limit. Values for parameters not determined were assigned non-zero values using (in order of preference): (1) the replicate determination value (samples with designation "R" in the name in Table 1, or (2) the value of a non-replicate determination made at the same site (e.g., 00-LL1 and 99-LL1). Geochemical data were log-transformed (with the exception of pH) to minimize the significance of unit differences and the high degree of variation in variable values among samples. Prior to running the PCA, the elements of each cross-products matrix were "correlated" (centered and standardized by standard deviation) to remove magnitude differences among variables [37]. The number of statistically significant principal components was assessed using the techniques previously described, as well as matrix randomization tests, in which the probability of attaining an equal or greater magnitude for each eigenvector is assessed by performing PCAs on data matrices in which the elements have been shuffled randomly.

PCA of the water data identified 3 principal components out of 12 total, with a 99.9% probability that their weights were non-random. PCA of solids data identified only 2 principal components out of 12, with 99% probability that their weights were non-random.

### Preparation of EXAFS Model Compounds

Details of sample preparation and procedures for XAFS data collection have been described previously [20,22,53]. Phases containing As in stoichiometric compounds were ground, and diluted with boron nitride prior to XAFS data collection in transmission mode. Samples containing As below 1 wt% were prepared as follows: the arsenian pyrite sample was hand picked from a pyrite-rich coal, ground, and mixed with mineral oil to minimize oxidation. As^5+ ^sorbed to Fe^3+ ^(hydr)oxide (mineralogy not determined) was prepared as described in [22], and the wet paste used for data collection. Solution samples of As^3+ ^and As^5+ ^oxoanions were prepared by dissolving the respective sodium salts in doubly deionized water and adjusting the pH with hydrochloric acid or sodium hydroxide to the desired value (near-neutral for both). The models with < 1% As were loaded into 3 mm-thick Teflon cells sealed on both sides by Kapton tape and XAFS spectra collected in fluorescence mode.

#### Preparation of Lava Cap/Lost Lake Samples

Ore samples were subsampled from archived hand specimens, shattered in a steel mortar and pestle, and ground dry to a fine powder in a SiC mortar and pestle. Grab samples of wet or dry tailings and microbial mats/flocs (Table 2) were stored at 4°C or -20°C until data collection. Core muds from Lost Lake were subsampled in a N_2_-filled glove bag and stored at -20°C until data collection. Samples were prepared for XAFS data collection under ambient conditions (tailings and microbial mats) or in an N_2_-filled glove bag (core mud samples). Excess water was removed from microbial mats/flocs by either centrifuging/decanting, or wicking on filter paper. A subsample of 00-LC1-m1 (a cyanobacterial/algal slime) was further rinsed in an excess of DDI water and centrifuged for analysis as sample 00-LC1-M2. Subsamples of the dewatered microbial slimes/flocs, the grab tailings samples, and the core muds were hand-homogenized using a prewashed spatula, then loaded into 3 mm telfon cells sealed on both sides by Kapton tape.

#### XAFS Data Collection, Reduction and Analysis

XAFS data were collected at the Stanford Synchrotron Radiation Laboratory (beamlines IV-3 and II-3) in transmission mode for diluted model compounds, and fluorescence mode for all other models and samples as described in [14,20-22]. Data were collected under ambient conditions, after examination of test scans showing no beam-induced oxidation or reduction of As in these samples [53]. The typical procedures of XAFS data reduction were followed in this study, and consist of averaging successive data scans, calibration to the energy of As(0) foil at 11,867.0 eV [54], background removal using linear or Gaussian functions, normalization to the "free atom" absorbance (i.e., Fermi level) at 11,885.0 eV and extraction of the extended XAFS (EXAFS) portion of the spectrum. An overview of these procedures can be found in [55], and details of implementation specific for As in natural materials can be found in [14,20-22]. The data reduction programs EXAFSPAK [56] and SixPack v.0.53 [57] were used to execute these procedures. X-ray absorption near-edge spectra (XANES) were not used for analysis, because spectra of adequate resolution could not be obtained under the data collection conditions employed. Note that the lowered resolution has no effect on the features in the EXAFS region of the X-ray absorption spectrum.

PCA of XAFS spectra was performed using SixPack v 0.53. The operations of PCA for spectra and definition of terms were described previously [36,38]. After interpolation of calibrated, normalized spectral data to a uniform energy grid, a data matrix was constructed composed of *c *sample columns and *r *rows of energy points (the value for *r *depends on the energy range specified). The number of PCs was assessed using the Malinowski indicator function IND [58], the amount of variance explained by each component, and the requirement that all sample spectra in the set could be completely reconstructed using the selected PCs. Due to strong chemical dissimilarities, samples were separated into two groups: ore, tailings and tailings-rich Lost Lake shallow core mud were place in group 1 (n = 11); microbial mats/flocs/water filtrate samples were placed in group 2 (n = 7; Table 2). Matrix randomization tests are not available in SixPack v. 0.53, so sensitivity tests were performed in which the results were compared to those obtained when a sample spectrum was removed at random from the set. It was determined that Group 2 PCA results were robust only when EXAFS data were used in unweighted form, but group 1 PCA results were robust under k^3^-weighted and unweighted conditions. Weighting the spectra by an exponent of k emphasizes the contribution of more distant scatters to the EXAFS spectrum, which aids in their analysis using either the shell-by-shell fitting or the linear combination, least-squares approaches. k-weighting unfortunately also amplifies the contribution of noise to spectra with low signal-to-noise ratio [55]. Based on these considerations, the PCA for group 1 was performed on k^3^-weighted EXAFS spectra, and the group 2 PCA on unweighted EXAFS spectra.

Target transformation (TT) was performed on spectra representing possible As species present in the samples in order to identify those best suited for use as models in LCLS analysis of sample spectra (also performed using SixPack v 0.53). The spectral library used for TT included the following compounds: As^5+^sorbed to Fe^3+ ^(hydr)oxide, arseniosiderite (CaFe_3_(AsO_4_)_3_O_2_·3H_2_O), aqueous As^5+^, amorphous FeAsO_4_, aqueous As^3+^, arsenate-substituted K-jarosite [KFe_3_(SO_4_,AsO_4_)_2_(OH)_6_], calcium arsenate [Ca_3_(AsO_4_)_2_], scorodite (FeAsO_4 _2H_2_O), arsenopyrite (FeAsS), arsenian pyrite [Fe(As,S)_2_], synthetic arsenic sulfide (As_2_S_3_), and natural orpiment (As_2_S_3_). Plots of most of these model compounds are available in Additional File 3. For LC analysis, small shifts in momentum (k) space were enabled for the sample spectrum only. Fit components were forced to be positive, but their sum was not forced to equal one. The accuracy of LC has previously been estimated to be 5-10% based on analysis of physical mixtures of As-bearing compounds [53].

## Abbreviations

**Alk**: alkalinity; **b.d**.: below detection; **DO**: dissolved oxygen; **EXAFS**: extended X-ray absorption fine structure spectroscopy; **HG-CT-GC-AAS**: Hydride generation, cryogenic-trapping, gas chromatographic column separation and analysis by atomic absorption spectroscopy; **HG-CT-GC-AFS**: Hydride generation-cryogenic trapping-gas chromatographic column separation and analysis by atomic fluorescence spectroscopy; **IC**: ion chromatography; **LCLS**: linear combination, least-squares fitting; **LCMS**: Lava Cap Mine Superfund Site; **n.d**.: not determined; **n.r**: not reported; **PC**: principal component; **PCA**: principal component analysis; **SpC**: specific conductivity; **XAFS**: X-ray absorption fine structure spectroscopy; **XANES**: X-ray absorption near-edge spectra; **TT**: target transformation

## Competing interests

The authors declare that they have no competing interests.

## Authors' contributions

ALF assisted in collection of water samples, and collected/analyzed about half of the solid samples. XAFS data collection and interpretation was performed by ALF. ALF was the primary author of all but the background sections of this manuscript. RPA led the collection and analysis of water samples, was responsible for the chemical analysis of about 1/2 of the solid samples, and was the primary author of the initial draft of the background section of the manuscript. JJR provided guidance and oversight as chief of the USGS-funded project *Geoenvironmental Impacts of Mercury and As*, which provided financial support for this work. All authors have read and approved the final manuscript.

## Supplementary Material

Additional File 1 **Electron micrograph images of ore sample and biogenic iron (hydr)oxide from the Lava Cap Mine**. Left panel: Backscattered electron image of pyrite-rich ore sample LCD3, illustrating major sulfide phases including arsenopyrite. Right panel: scanning electron micrograph of biogenic Fe^3+ ^(hydr)oxide floc of the type common at the LCMS. Iron (hydr)oxide precipitates as ca. 100 nm balls and coats (1) tubular sheaths characteristic of the *Sphaerotilus-Leptothrix *group of bacteria as well as (2) a cluster of rod-shaped (putative) bacteria external to the sheath. Click here for file

Additional File 2 **Arseniosiderite solubility estimates**. Water data from Table 1 are used to estimate the saturation state of waters with respect to arseniosiderite using the solubility expression of Krause and Ettel [51]. Click here for file

Additional File 3 **Model Compound EXAFS Spectra**. Normalized, k^3^-weighted EXAFS spectra [χ(k)*k^3^)] of As model compounds tested in PCA target transformations and used in non-linear least squares fits. Spectra representing As highly-ordered coordination environments are plotted in (a), and spectra representing As in less-ordered coordination environments are presented in (b). Click here for file
