# Supplementary figures and images for: Arsenic species in weathering mine tailings and biogenic solids at the Lava Cap Mine Superfund Site, Nevada City, CA
Source: Geochem Trans. 2011 Jan 24;12:1. doi: 10.1186/1467-4866-12-1 (PMC3037876; doi:10.1186/1467-4866-12-1)

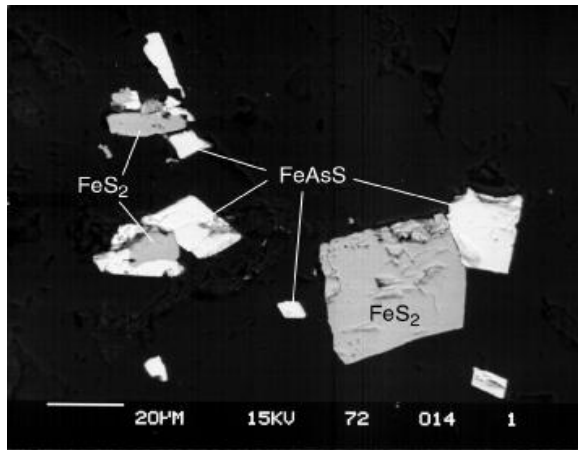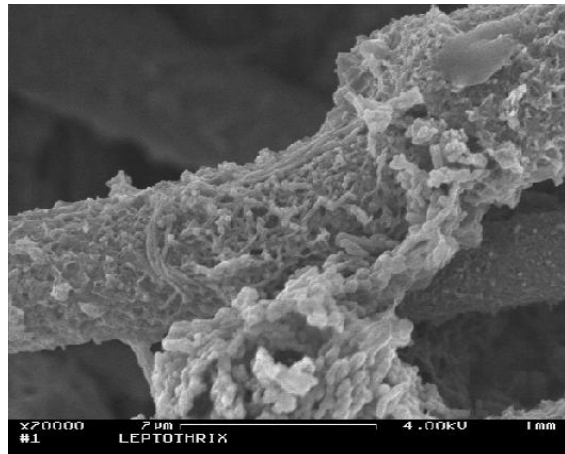

Supplement: Additional File 1 — Electron micrograph images of ore sample and biogenic iron (hydr)oxide from the Lava Cap Mine. Left panel: Backscattered electron image of pyrite-rich ore sample LCD3, illustrating major sulfide phases including arsenopyrite. Right panel: scanning electron micrograph of biogenic Fe3+ (hydr)oxide floc of the type common at the LCMS. Iron (hydr)oxide precipitates as ca. 100 nm balls and coats (1) tubular sheaths characteristic of the Sphaerotilus-Leptothrix group of bacteria as well as (2) a cluster of rod-shaped (putative) bacteria external to the sheath. [file 1467-4866-12-1-S1.PDF]

## Crystalline models

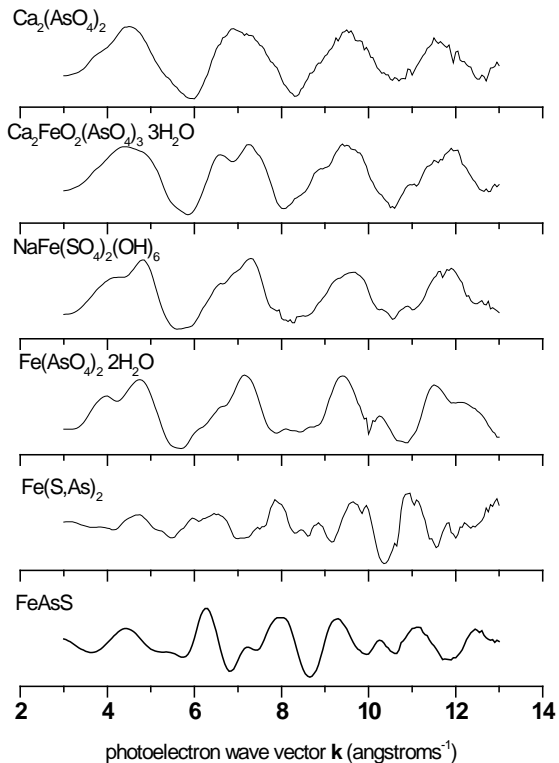

## Aqueous and sorbed models

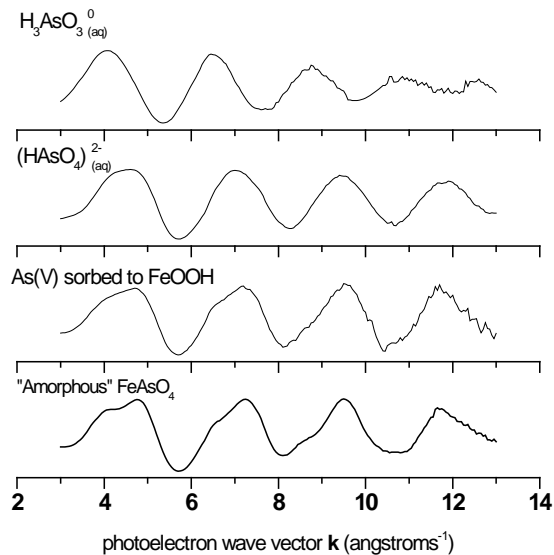

Supplement: Additional File 3 — Model Compound EXAFS Spectra. Normalized, k3-weighted EXAFS spectra [χ(k)*k3)] of As model compounds tested in PCA target transformations and used in non-linear least squares fits. Spectra representing As highly-ordered coordination environments are plotted in (a), and spectra representing As in less-ordered coordination environments are presented in (b). [file 1467-4866-12-1-S3.PDF]
