# Supplementary material for: Arsenic species in weathering mine tailings and biogenic solids at the Lava Cap Mine Superfund Site, Nevada City, CA
Source: Geochem Trans. 2011 Jan 24;12:1. doi: 10.1186/1467-4866-12-1 (PMC3037876; doi:10.1186/1467-4866-12-1)
Supplement: Additional File 2 — Arseniosiderite solubility estimates. Water data from Table 1 are used to estimate the saturation state of waters with respect to arseniosiderite using the solubility expression of Krause and Ettel [51]. [file 1467-4866-12-1-S2.PDF]

Sample ID	pH	[H]^4	Fe total	As total inorganic	Ca	Fe tot	As tot, inorg	Ca	IAP arseniosiderite	IAP/KSP	Saturated?	IAP/KSP	saturated
**Units–**	-log[H+]		mg/L	µg/L	mg/l	Molar	molar	molar	activity = concentrat	ksp=1.87 E-10		ksp = 4.74E-15	
**method**	electrode		Ferrozine (spectroph	HG-CT-GC-	ICP								
**confidence**	± 0.1		n.d.	± 5%	± 5%								
**LCD**													
99	7.3	6.3096E-30	0.719	459	66.8	1.2875E-05	6.1265E-06	0.0016667	2.16074E-07	1.155E+03	yes	4.559E+07	yes
00	7.2	1.5849E-29	0.769	355	65.3	1.377E-05	4.7383E-06	0.0016293	4.6528E-08	2.488E+02	yes	9.816E+06	yes
**Lost Lake Sites**													
**LL5**													
99	8.5	1E-34	0.451	66.8	7.15	8.0759E-06	8.9161E-07	0.0001784	1.18822E-07	6.354E+02	yes	2.507E+07	yes
99-PW[6]	7	1E-28	10.7	3250	26.3	0.0001916	4.3379E-05	0.0006562	0.002472505	1.322E+07	yes	5.216E+11	yes
00	9.4	2.5119E-38	0.088	17.9	2.9	1.5758E-06	2.3892E-07	7.236E-05	1.11231E-08	5.948E+01	yes	2.347E+06	yes
00-PW	n.d.	#VALUE!	0.845	45.1	22	1.5131E-05	6.0197E-07	0.0005489	#VALUE!				
00-R[8]	7	1E-28	0.399	36.8	11.3	7.1448E-06	4.9118E-07	0.000282	3.43592E-14	1.837E-04	no	7.249E+00	yes
**LL6**													
99	8.6	3.9811E-35	0.401	86.7	6.8	7.1806E-06	1.1572E-06	0.0001697	4.1489E-07	2.219E+03	yes	8.753E+07	yes
00-R	8.5	1E-34	0.51	44.1	8.2	9.1324E-06	5.8862E-07	0.0002046	6.50247E-08	3.477E+02	yes	1.372E+07	yes
99-PW	7.2	1.5849E-29	7.01	31.3	n.d.	0.00012553	4.1777E-07	#VALUE!					
00-PW	n.d.	#VALUE!	0.923	38.1	31	1.6528E-05	5.0854E-07	0.0007735	#VALUE!				
**LL12**													
99	8	1E-32	0.379	65.8	6.55	6.7866E-06	8.7826E-07	0.0001634	5.65591E-10	3.025E+00	yes	1.193E+05	yes
99-PW	7	1E-28	16.5	1580	32.4	0.00029546	2.1089E-05	0.0008084	0.00158102	8.455E+06	yes	3.335E+11	yes
00	7.1	3.9811E-29	0.075	9.08	2.8	1.343E-06	1.2119E-07	6.986E-05	5.28666E-19	2.827E-09	no	1.115E-04	no
00-PW	n.d	#VALUE!	7.8	1090	48	0.00013967	1.4549E-05	0.0011977	#VALUE!				
00-R	7.3	6.3096E-30	0.406	36.1	9.05	7.2701E-06	4.8184E-07	0.0002258	3.47393E-13	1.858E-03	no	7.329E+01	yes
00-R-PW	7.7	1.5849E-31	0.859	2050	34	1.5382E-05	2.7362E-05	0.0008483	0.00033855	1.810E+06	yes	7.142E+10	yes
**Pond In tailings**													
**LL2**													
99	7.8	6.3096E-32	0.008	440	65.5	1.4325E-07	5.8729E-06	0.0016343	2.52078E-11	1.348E-01	no	5.318E+03	yes
99-R	8.1	3.9811E-33	0.008	1340	59.5	1.4325E-07	1.7886E-05	0.0014846	9.31199E-09	4.980E+01	yes	1.965E+06	yes
00	7.6	3.9811E-31	0.018	614	59.1	3.2232E-07	8.1953E-06	0.0014746	1.00675E-10	5.384E-01	no	2.124E+04	yes
00-R	7.6	3.9811E-31	0.009	533	62.7	1.6116E-07	7.1142E-06	0.0015644	9.26549E-12	4.955E-02	no	1.955E+03	yes
**Seep at base of Lost**													
**LL1**													
98	6.7	1.5849E-27	5.74	78.6	19.6	0.00010278	1.0491E-06	0.000489	1.89208E-10	1.012E+00	yes	3.992E+04	yes
99	6.2	1.5849E-25	0.053	1.1	7.9	9.4906E-07	1.4682E-08	0.0001971	6.63261E-25	3.547E-15	no	1.399E-10	no
99-R	6.5	1E-26	5.53	92.4	18.7	9.9024E-05	1.2333E-06	0.0004666	3.96551E-11	2.121E-01	no	8.366E+03	yes
99-R2	6.5	1E-26		92.4	19.2	9.222E-05	1.2333E-06	0.0004791	3.37648E-11	1.806E-01	no	7.123E+03	yes
			5.15										
00	6.7	1.5849E-27	6.251	61.8	20.5	0.00011193	8.2487E-07	0.0005115	1.29941E-10	6.949E-01	no	2.741E+04	yes
00-R	6.6	3.9811E-27	5.77	85.1	21.7	0.00010332	1.1359E-06	0.0005414	1.19031E-10	6.365E-01	no	2.511E+04	yes
**LL10**													
99	7	1E-28	1.77	43.2	19.7	3.1695E-05	5.7661E-07	0.0004915	1.47478E-11	7.887E-02	no	3.111E+03	yes
**Background**													
**LL8**													
98	7	1E-28	0.005	0.098	3.4	8.9534E-08	1.308E-09	8.483E-05	1.15603E-28	6.182E-19	no	2.439E-14	no
99	6.5	1E-26	b.d.	0.091	3.1	#VALUE!	1.2146E-09	7.735E-05	#VALUE!				
00-R	6.8	6.3096E-28	b.d	0.159	3.3	#VALUE!	2.1222E-09	8.234E-05	#VALUE!				
